# Supplementary figures and images for: Plasmodium falciparum K13 mutations in Africa and Asia impact artemisinin resistance and parasite fitness
Source: eLife. 2021 Jul 19;10:e66277. doi: 10.7554/eLife.66277 (PMC8321553; doi:10.7554/eLife.66277)

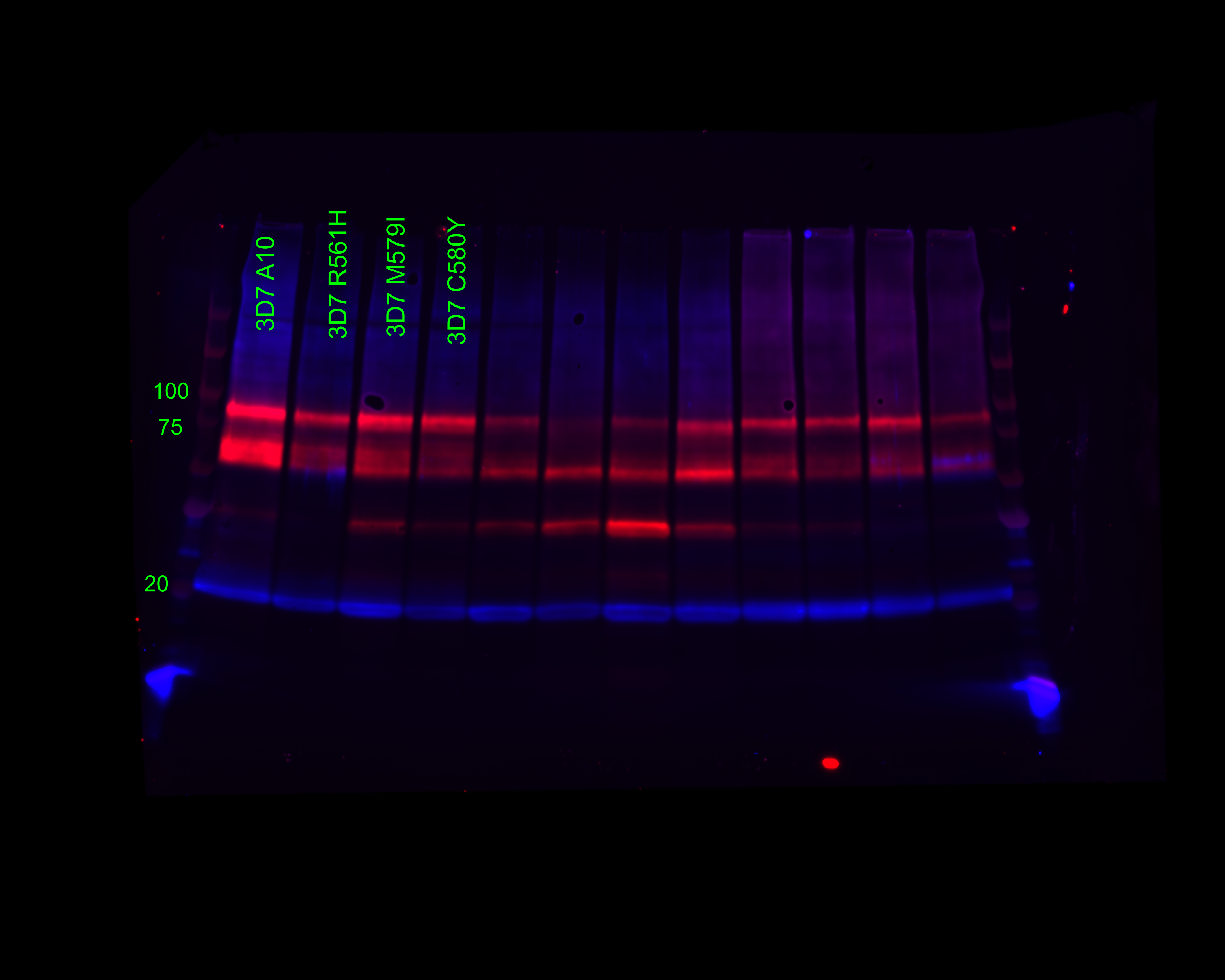

Supplement: Figure 2—figure supplement 1—source data 1. [file elife-66277-fig2-figsupp1-data1.zip › Figure 2ΓÇôfigure supplement 1ΓÇôsource data 1/BioRep-1.png]

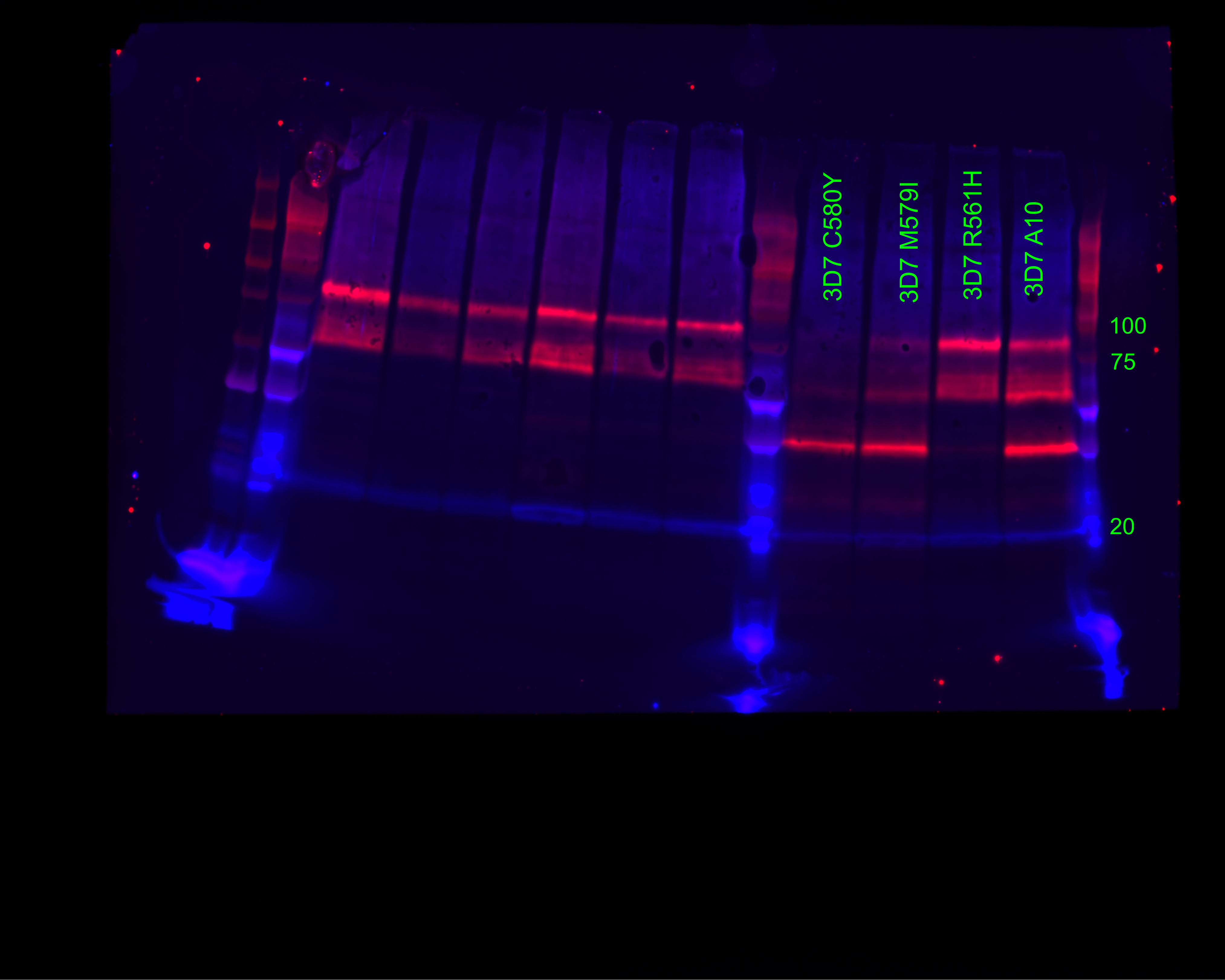

Supplement: Figure 2—figure supplement 1—source data 1. [file elife-66277-fig2-figsupp1-data1.zip › Figure 2ΓÇôfigure supplement 1ΓÇôsource data 1/BioRep-2.png]

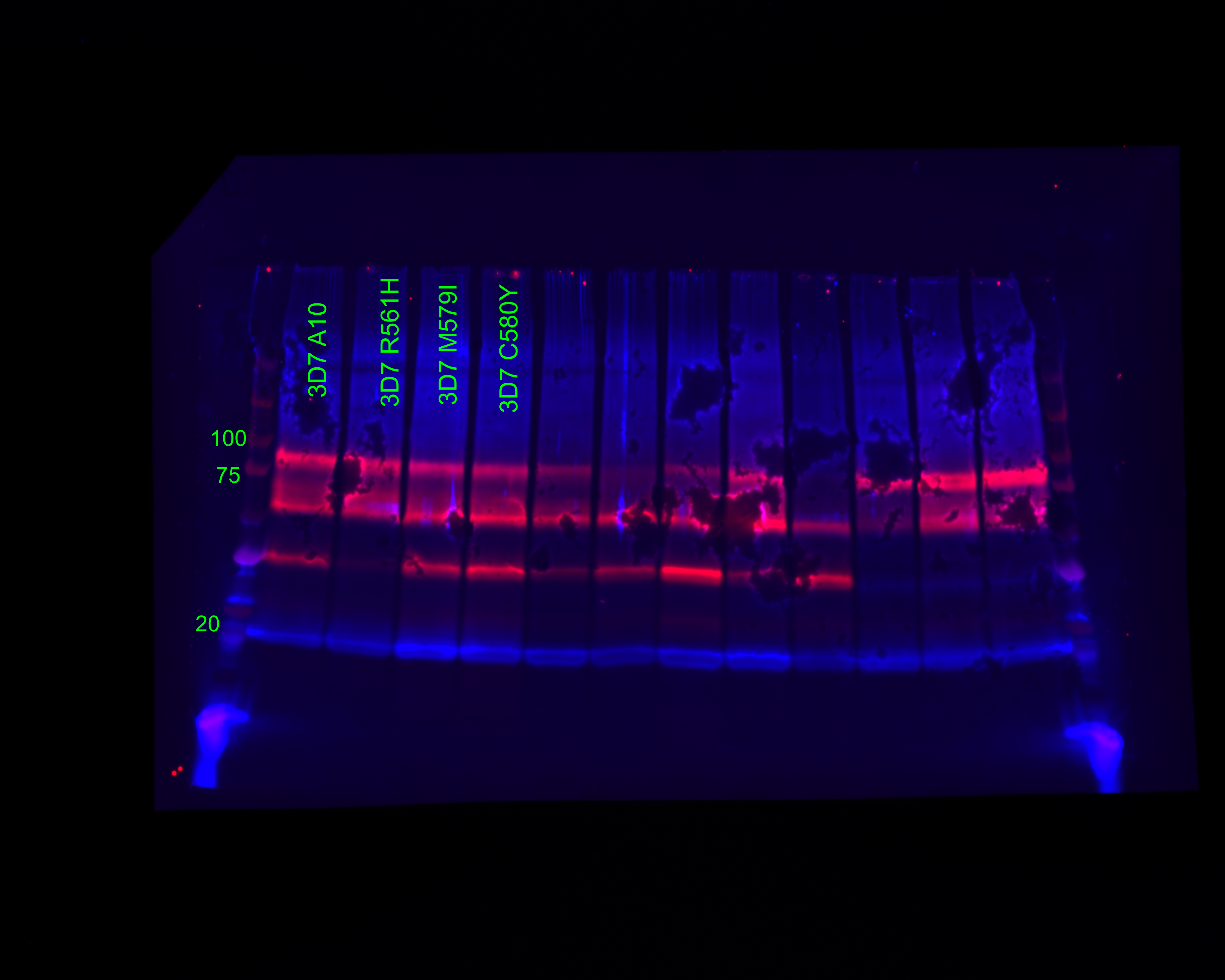

Supplement: Figure 2—figure supplement 1—source data 1. [file elife-66277-fig2-figsupp1-data1.zip › Figure 2ΓÇôfigure supplement 1ΓÇôsource data 1/BioRep-3.png]

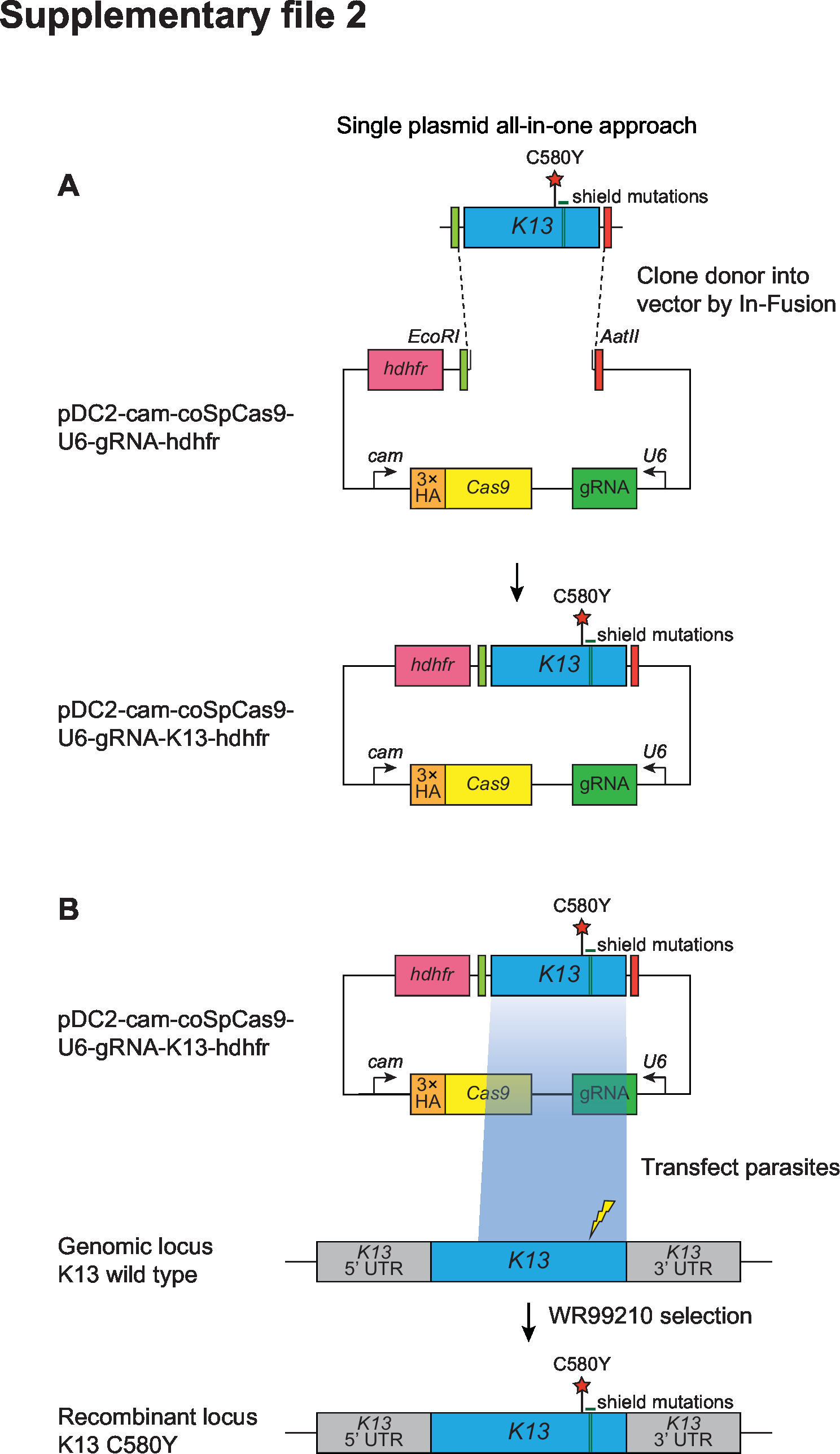

Supplement: Supplementary file 2. — All-in-one plasmid approach used for CRISPR/Cas9-mediated K13 gene editing, consisting of a K13-specific donor template for homology-directed repair, a K13-specific gRNA expressed from the U6 promoter, a Cas9 cassette with expression driven by the calmodulin (cam) promoter, and a selectable marker (human dhfr, conferring resistance to the antimalarial WR99210 that inhibits P. falciparum DHFR). The Cas9 sequence was codon-optimized for improved expression in P. falciparum. Donors coding for specific mutations of interest (e.g., K13 C580Y, red star) were generated by site-directed mutagenesis of the K13 wild-type donor sequence. Green bars indicate the presence of silent shield mutations that were introduced to protect the edited locus from further cleavage. The lightning bolt indicates the location of the cut site in the genomic target locus. Primers used for cloning and final plasmids are described in Supplementary files 7 and 8, respectively. [file elife-66277-supp2.jpg]

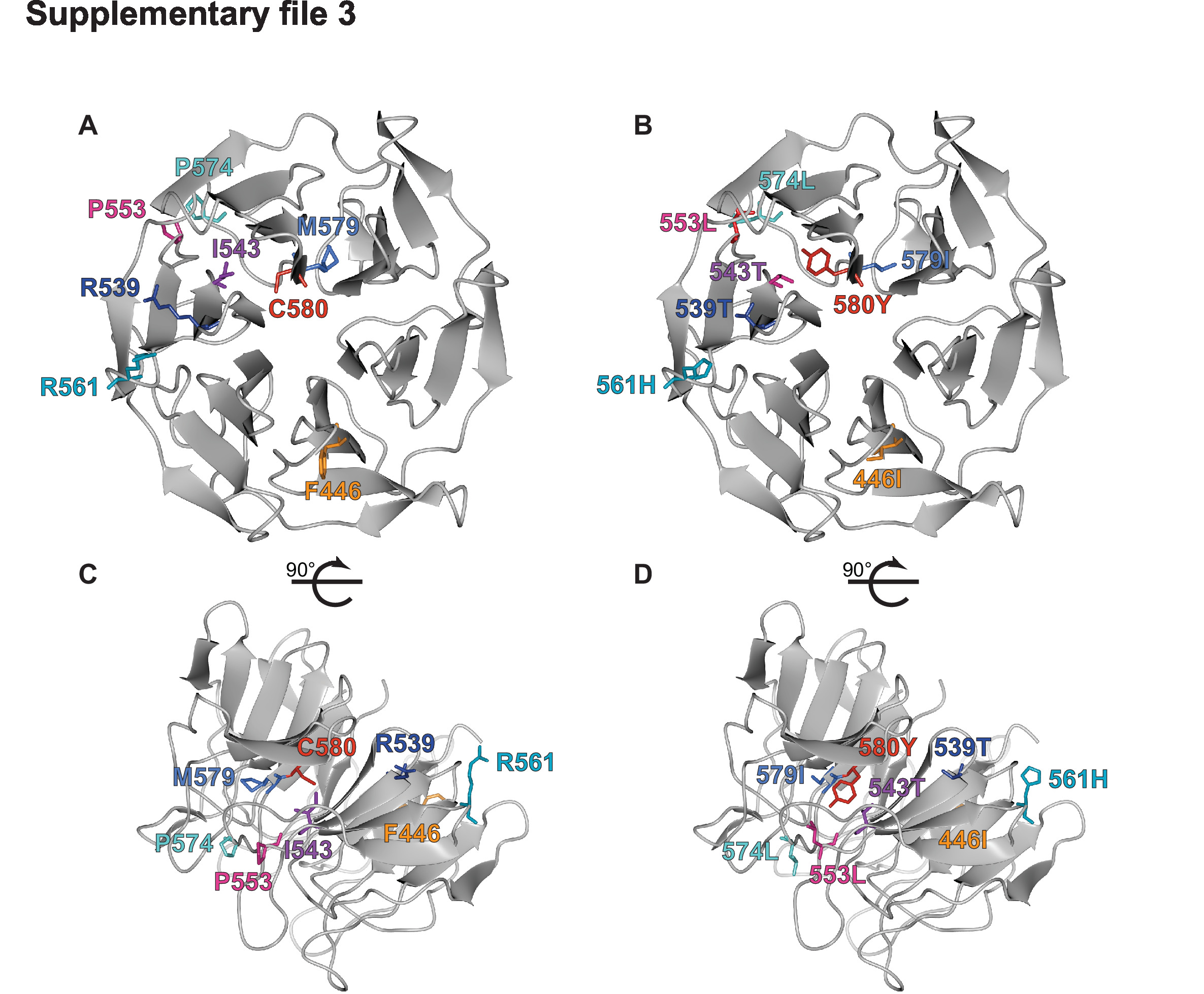

Supplement: Supplementary file 3. — (A, B) Top and (C, D) side views of the crystal structure of the K13 propeller domain (PDB ID: 4YY8), highlighting residues of interest (F446I, orange; R539T, dark blue; I543T, purple; P553L, pink; R561H, dark turquoise; P574L, light turquoise; M579I medium blue; C580Y, red). Structures shown in (A) and (C) show wild-type residues while (B) and (D) show mutated residues. [file elife-66277-supp3.jpg]

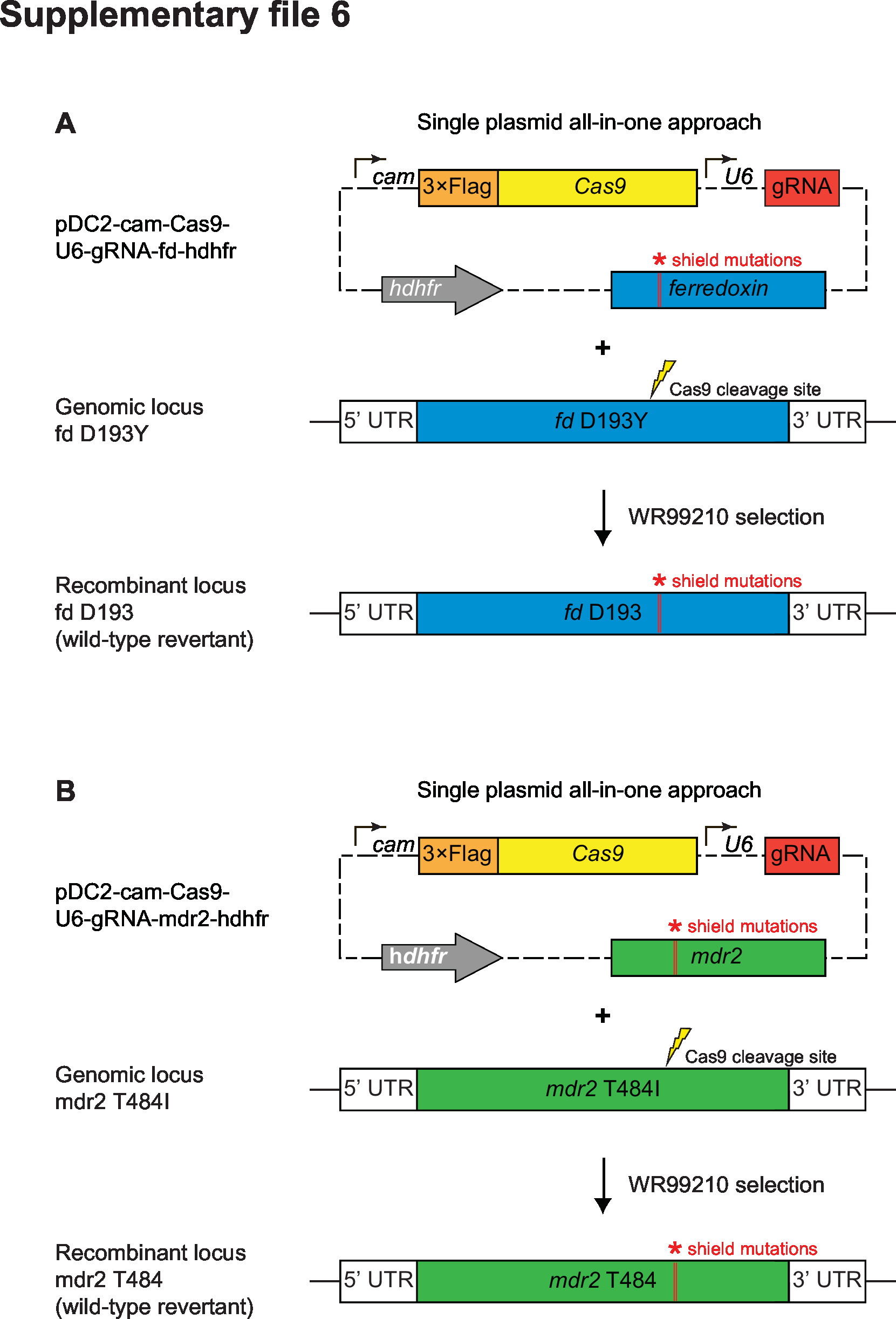

Supplement: Supplementary file 6. — All-in-one plasmid approaches used for CRISPR/Cas9-mediated editing of (A) the ferredoxin (fd) locus or (B) the multidrug resistance protein 2 (mdr2) locus. Plasmids consisted of a (A) fd or (B) mdr2 specific donor template for homology-directed repair, a gene-specific gRNA expressed from the U6 promoter, a Cas9 cassette with expression driven by the cam promoter, and a selectable marker (human dhfr, conferring resistance to WR99210). Donors coding for specific mutations of interest (fd D193Y or mdr2 T484I) were generated by site-directed mutagenesis of the wild-type donor sequences. Red bars indicate the presence of silent shield mutations used to protect edited loci from further cleavage. Primers used for cloning and final plasmids are described in Supplementary files 7 and 8, respectively. [file elife-66277-supp6.jpg]
