# Supplementary material for: Plasmodium falciparum K13 mutations in Africa and Asia impact artemisinin resistance and parasite fitness
Source: eLife. 2021 Jul 19;10:e66277. doi: 10.7554/eLife.66277 (PMC8321553; doi:10.7554/eLife.66277)
Supplement: Transparent reporting form [file elife-66277-transrepform.docx]

***eLife’s* transparent reporting form**

We encourage authors to provide detailed information *within their submission* to facilitate the interpretation and replication of experiments. Authors can upload supporting documentation to indicate the use of appropriate reporting guidelines for health-related research (see [EQUATOR Network](http://www.equator-network.org/%20)), life science research (see the [BioSharing Information Resource](https://biosharing.org/)), or the [ARRIVE guidelines](http://www.plosbiology.org/article/info:doi/10.1371/journal.pbio.1000412) for reporting work involving animal research. Where applicable, authors should refer to any relevant reporting standards documents in this form.

If you have any questions, please consult our Journal Policies and/or contact us: [editorial@elifesciences.org](mailto:editorial@elifesciences.org).

**Sample-size estimation**

- You should state whether an appropriate sample size was computed when the study was being designed
- You should state the statistical method of sample size computation and any required assumptions
- If no explicit power analysis was used, you should describe how you decided what sample (replicate) size (number) to use

Please outline where this information can be found within the submission (e.g., sections or figure legends), or explain why this information doesn’t apply to your submission:

Unpaired *t* tests were used to compare *Plasmodium falciparum* parasite lines that had been engineered to express different *K13* alleles. The *t* test assumes a normal distribution and is appropriate when using a small sample size and unknown standard deviations for each population. These tests were conducted to assess for statistically significant differences between lines in data presented in Figures 2, 5 and 6.

The *t* test is a standard in our research field, as exemplified by its use in our recent *eLife* article by Mathieu et al. (2020, PMID 32394893), as well as methodologically related articles by Lamonte et al. (2020 Nature Commun, PMID 32286267), Harding et al. (2020, Nature Commun, PMID 32968076) and Paquet et al. (2017, Science Transl Med, PMID 28446690).

Results for each line and data point in these figures were collated from 2 to 13 independent repeats, as designated in our legends to Figures 2, 5, 6, and 7 (there were no statistically significant differences between lines in Figure 7). A standard for our field is 2 or more independent repeats.

**Replicates**

- You should report how often each experiment was performed
- You should include a definition of biological versus technical replication
- The data obtained should be provided and sufficient information should be provided to indicate the number of independent biological and/or technical replicates
- If you encountered any outliers, you should describe how these were handled
- Criteria for exclusion/inclusion of data should be clearly stated
- High-throughput sequence data should be uploaded before submission, with a private link for reviewers provided (these are available from both GEO and ArrayExpress)

Please outline where this information can be found within the submission (e.g., sections or figure legends), or explain why this information doesn’t apply to your submission:

Figures 2, 5, 6 and 7 have legends that list the numbers of independent repeats as 3 to 8, 3 to 13, 3 to 7, and 2 to 3, respectively. Each figure legend indicates that repeats were performed with technical duplicates.

Means, SEM values and numbers of independent repeats for these data sets are presented in Figure 2–source data 1, Figure 3–source data 1, Figure 5–source data 1, Figure 5–source data 2, Figure 6–source data 1, Figure 7–source data 1, Figure 7–source data 2, and Figure 7–source data 3. All data from these quantitative assays were included and there was no requirement for criteria to include or exclude assays.

**Statistical reporting**

- Statistical analysis methods should be described and justified
- Raw data should be presented in figures whenever informative to do so (typically when N per group is less than 10)
- For each experiment, you should identify the statistical tests used, exact values of N, definitions of center, methods of multiple test correction, and dispersion and precision measures (e.g., mean, median, SD, SEM, confidence intervals; and, for the major substantive results, a measure of effect size (e.g., Pearson's r, Cohen's d)
- Report exact p-values wherever possible alongside the summary statistics and 95% confidence intervals. These should be reported for all key questions and not only when the p-value is less than 0.05.

Please outline where this information can be found within the submission (e.g., sections or figure legends), or explain why this information doesn’t apply to your submission:

Legends to Figures 2, 5 and 6 indicate the type of statistical test and refer to the supplemental tables where means, SEMs, numbers of independent repeats, and P values are listed. Figures 2, 5, 6 and 7 show data as means ± SEM and also show the individual repeats from each independent experiment.

(For large datasets, or papers with a very large number of statistical tests, you may upload a single table file with tests, Ns, etc., with reference to sections in the manuscript.)

**Group allocation**

- Indicate how samples were allocated into experimental groups (in the case of clinical studies, please specify allocation to treatment method); if randomization was used, please also state if restricted randomization was applied
- Indicate if masking was used during group allocation, data collection and/or data analysis

Please outline where this information can be found within the submission (e.g., sections or figure legends), or explain why this information doesn’t apply to your submission:

Not applicable.

**Additional data files (“source data”)**

- We encourage you to upload relevant additional data files, such as numerical data that are represented as a graph in a figure, or as a summary table
- Where provided, these should be in the most useful format, and they can be uploaded as “Source data” files linked to a main figure or table
- Include model definition files including the full list of parameters used
- Include code used for data analysis (e.g., R, MatLab)
- Avoid stating that data files are “available upon request”

Please indicate the figures or tables for which source data files have been provided:

All data used to generate Figures are already collated as source data provided as supplementary information.
